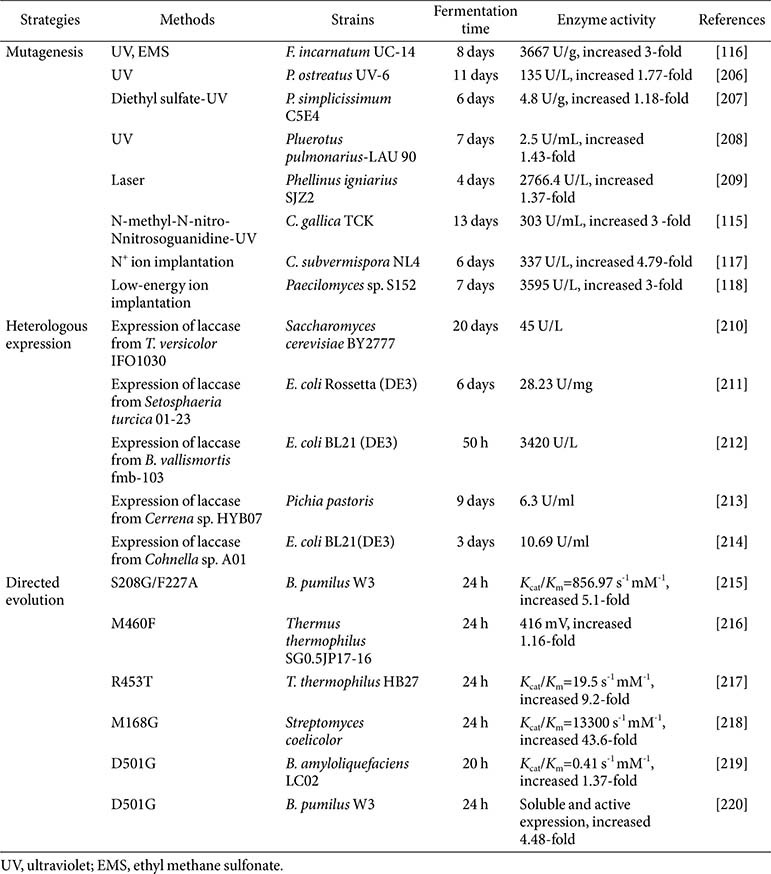# Recent Advances in Laccase Production: Challenges and Future Perspectives

**DOI:** 10.4014/jmb.2507.07052

**Published:** 2025-10-27

**Authors:** Mingxia Jiao, Wenhu Chen, Xiangling Jiang, Di Huang, Yi Jiang, Hongling Liu, Haibo Yuan, Tengfei Wang

**Affiliations:** 1 State Key Laboratory of Green Papermaking and Resource Recycling, Qilu University of Technology (Shandong Academy of Sciences), Jinan 250353, Shandong, P.R. China; 2 Key Laboratory of Shandong Microbial Engineering, School of Bioengineering, Qilu University of Technology (Shandong Academy of Sciences), Jinan 250353, Shandong, P.R. China

**Keywords:** Laccase, catalytic mechanism, laccase production

## Abstract

Laccase, a type of multicopper oxidase, exhibits several notable advantages, including broad distribution, wide catalytic scope, and high oxidation-reduction potential. Owing to its enormous potential in food, textiles, paper, chemical synthesis, and bioremediation, laccase is considered a green catalyst in wide-ranging industrial applications. To aid in understanding the current developments in laccase research, this review introduces the physiological functions, classification, and reaction mechanisms of laccase, with a focus on improvement strategies. In addition, the updated studies and advancements in laccase production are summarized. Finally, the possible research trends and general development directions for more efficient laccase production are also proposed.

## Introduction

Laccase (ρ-diphenol: dioxygen oxidoreductases; EC 1.10.3.2) is a multicopper oxidase belonging to the superfamily of multicopper oxidases (MCOs), a group of enzymes with diverse biological functions and substrate specificities [1]. MCOs can be classified into three types based on the number of cupredoxin-like domains: two-domain (2D), three-domain (3D), and six-domain (6D) [2, 3]. First discovered in the Japanese lacquer tree *Rhusvernicifera*, laccase was later found to exist extensively in fungi, higher plants, bacteria, algae, insects, and invertebrates [4-10]. Laccases can catalyze the oxidation of multitudinous compounds, including phenols, polyphenols, benzenethiols, amino-phenols, anilines, polyamines, hydroxyindoles, lignin, aryl diamines, and some inorganic ions [11].

**Fig. 1:**
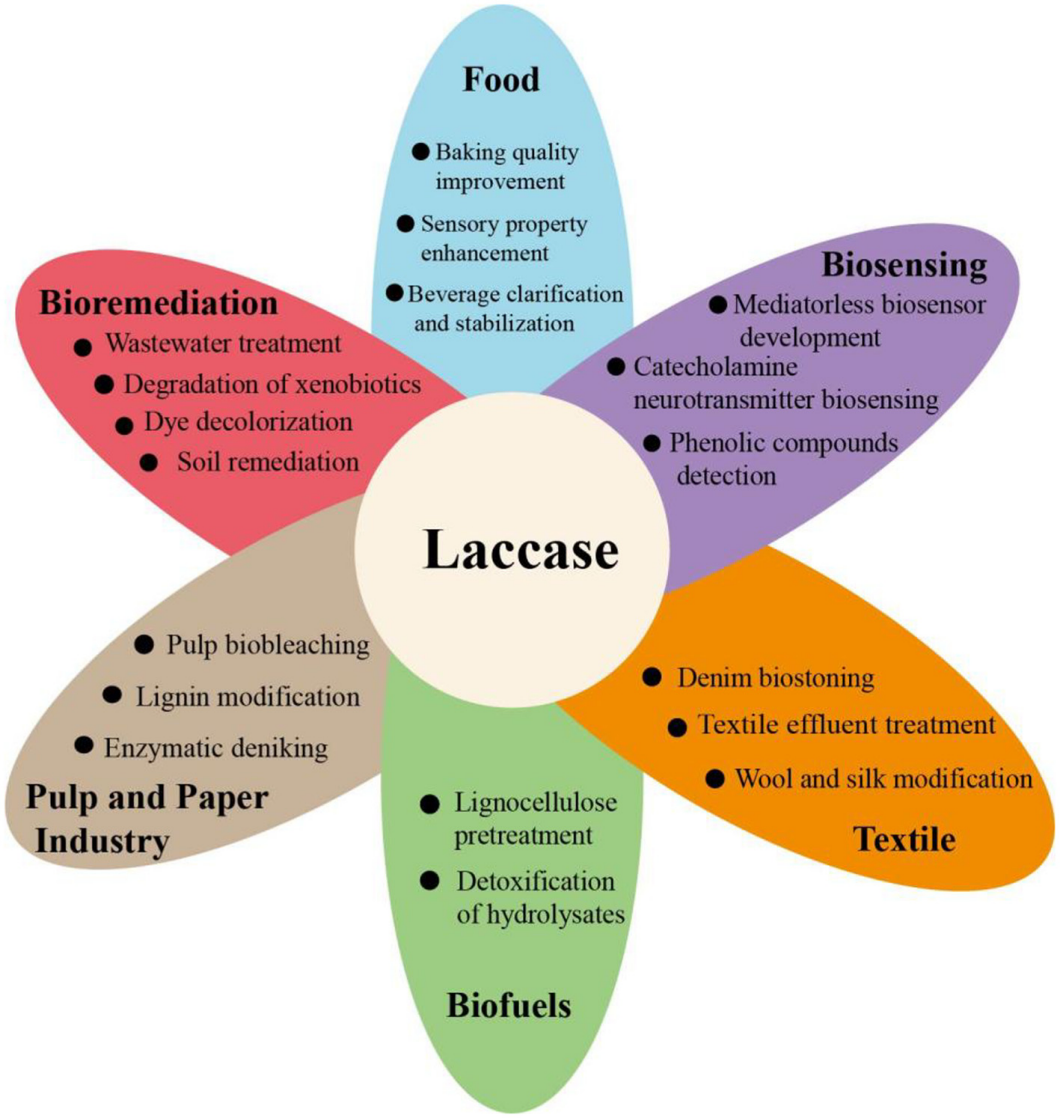
The industrial application fields of laccase.

The electrochemical potential is a significant property of laccases that varies between 0.3 and 0.8 V [12]. Compared to other laccases, fungal laccase has a larger redox potential [1]. However, bacterial laccases have received considerable attention because they show an obvious predilection for neutral and alkaline pH ranges, whereas laccases in fungi are preferred in the acidic pH range [13]. Owing to its broad range of substrates and the capacity to generate water as the only by-product, laccase is used in diverse industries, such as food, pulp and paper, biofuel, bioremediation, and biosensors [14] (Fig. 1).

Many recently reported studies on laccase have covered its industrial applications, modification based on biotechnology, action mechanism, and enzyme immobilization [14-17]. At present, however, despite an increasing demand for laccase production in numerous industries, the strategies regarding laccase production methods and their improvement have not been systematically summarized. To meet this need, we herein describe the characteristics of laccase, and then comprehensively summarize the latest achievements regarding methods and strategies for improving laccase production.

## Species, Characteristics, and Applications of Laccase

### Biological Function

The biological function of laccases depends on the life stage and source of the microorganisms involved [18]. For bacteria, most laccases are located intracellularly, such as *Bacillus subtilis* and *Azospirillum lipoferum* [19]. Laccase plays a vital role in cell pigmentation, spore protection, copper resistance, and/or electron transport [20-23]. For example, the outer spore-coat protein CotA participates in the synthesis of spore pigment, and hypothetically, it is responsible for most of the protection afforded by the *B. subtilis* spore coat against hydrogen peroxide and UV light [24]. Fungal laccases are involved in stress defense, pigment production, sporulation, plant pathogenesis, lignin degradation, and fruit body formation [25]. Additionally, laccases from plants are involved in plant biological processes, such as ion metabolism, abiotic stress protection, wound healing, polymerization of phenolic compounds, and maintenance of cell wall integrity [26-29]. Laccases have also been identified in various insects, and are mainly related to food detoxification and cuticle sclerotization [30]. The same functions of laccase in insects are also found in invertebrates. In addition, laccases help protect against bacterial pathogens in invertebrates, such as in *Litopenaeus vannamei* [31]. The role of laccases from algae includes degradation of lignocellulose, detoxification of phenolics in environments, and participation in synthesizing cell wall-associated polymers and UV-absorbing compounds [32, 33].

### Structure and Reaction Mechanism

Laccase is a blue oxidase glycoprotein with monomeric, dimeric, or tetrameric structures. Its protein molecule is composed of 10–45% carbohydrates and usually more than 500 amino acids [34]. The carbohydrate moiety (glycans) is composed of glucose, hexose amine, mannose, galactose, fructose, xylose, and/or arabinose. These carbohydrates are attached to the polypeptide of the enzyme by an N-linkage and differ according to the enzymés source [35]. It is evident that the carbohydrate moiety chains help define the structure of the enzyme by attaching to its molecule and causing a specific folding that has been shown to provide protective activities against proteolytic and extreme temperature degradation [35].

Generally, the catalytic center of laccase involves four copper ions: a T1-copper ion that determines the redox potential of laccase, a T2-copper ion, and double T3-copper ions that form a trinuclear copper cluster together [36]. Therefore, the laccases can be classified into four categories according to their T1 center (Table 1). Typical blue laccase was characterized by a 610 nm band in the UV–Vis spectra corresponding to the T1-copper center and a diminutive, hyperfine coupling constant in electron paramagnetic resonance (EPR) spectra [37]. Owing to the copper cysteine bond's absorption through ligand-to-metal charge transfer, the T1-copper ion gives the enzyme its striking blue color [38]. Additionally, it may freely interact with solvents including water, can be extracted from the enzyme molecule by different copper complexes, and be replaced by cobalt or mercury with a significant reduction in activity [39]. Each T3-copper ion coordinates three His residues, while each T2-copper ion coordinates two His residues and an oxygen atom as OH− [40]. A His-cysteine (Cys)-His tripeptide bridge connects the T1-copper and T2/3-copper ion and serves as an intramolecular electron transport route [41].

Some studies have reported that enzymes lacking the maximum peak at 600 nm in the UV–Vis spectra are called laccase-like enzymes because they have the same inherent catalytic activity as typical blue laccases. Meanwhile, another study reported that laccases lacking the blue copper atom are called “white” or “yellow” laccases [42]. According to current studies, however, the lack of a typical color in white laccases was attributed to the absence of T1 copper and possession of other non-copper atoms [43]; yellow laccases lack absorptions at 600 and 610 nm, and have metal ions, as do blue laccases [44]. Remarkably, the laccase from *Sclerotinia sclerotiorum*, referred to as a mixed blue-yellow laccase, has a T1 center that is non-detectable in UV–Vis spectra, but can be detected in the EPR spectrum, which demonstrates its resemblance to blue laccases [45]. In addition, some studies have reported that blue laccase can be artificially reduced to yellow laccase since it does not show an EPR spectrum or absorption at 600 nm [36].

**Fig. 2:**
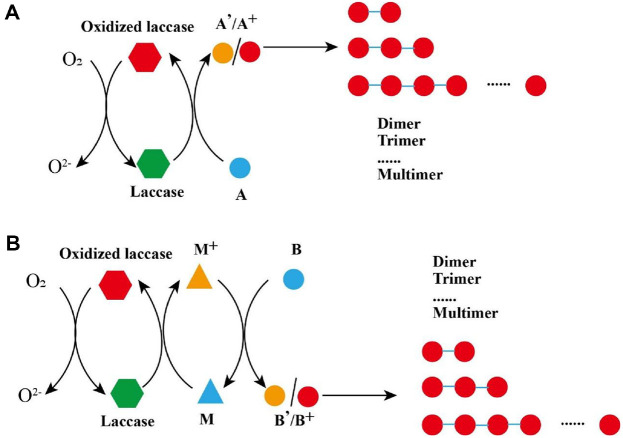
Schematic representation of reaction types catalyzed by laccase. (**A**) direct oxidation; (**B**) indirect oxidation. M, mediator; A, low-redox potential substrates; B, high-redox potential substrates; A' / B', stable oxide; M^+^ / A^+^ / B^+^, radicals.

The catalytic reactions of laccase can be divided into two types: direct oxidation and indirect oxidation. For direct oxidation, the substrate molecules are oxidized to the relevant radicals by direct interaction with the T1-copper in laccase (Fig. 2A) [49]. The substrates are oxidized as they release electrons to the T1-copper ion through a histidine residue. The electrons are transferred to the trinuclear copper cluster from the T1-copper ion, comprising one T2-copper ion and double T3-copper ions, where the oxygen molecules are bound and reduced to water [50]. For instance, in the biodegradation of polycyclic aromatic hydrocarbons, the benzo(*a*)anthracene group is directly oxidized via four steps by laccase in *Anthracophyllum*, and butanoic anhydride eventually forms [51]. The ether bond of 5-chloro-2-(2,4-dichlorophenoxy) phenol is broken due to the oxidation of laccase, while it is oxidized to phenoxyl radicals [52]. The bonds of the natural-sourced lignin can be separated by laccase, including C_α_-oxidation, aryl-alkyl, and C_α_-C_β_ cleavages [16].

As for the indirect reaction, the process starts with the substrate being oxidized to produce matching radicals, which can subsequently cause the substrate to repolymerize or depolymerize (Fig. 2B). Whether the radicals are the same or different, both can covalently couple to form oligo- or polymeric products [53]. The fungus *Daldinia eschscholzii* IFB-TL01 produces the unusually structured immunosuppressant’s (±)-dalesconols A, B, and C by laccase, with these unique characteristics being the result of promiscuous and atroposelective couplings of radicals derived from 1,3,6,8-tetrahydroxynaphthalene, 1,3,8-trihydroxynaphthalene, and 1,8-dihydroxynaphthalene, respectively [54]. There are also certain types of special radicals called mediators, which can oxidize other substrates after being oxidized by laccase. Three laccase-mediator system reaction mechanisms have been reported [55], including one that occurs via electron transfer of 2,2’-azino-bis(3-ethylbenzothiazoline-6-sulfonic-acid) (ABTS), in which an electron is lost to laccase and an electron is gained from substrates [56]. Another occurs via ionic oxidation, which is suggested in the case of 2,2,6,6-tetramethylpiperidinoxyl (TEMPO) and 2-azaadamantane N-oxyl (AZADO). The third happens via a hydrogen atom transfer, as suggested for 1-hydroxybenzotriazole (HBT), 2-hydroxybutyl acrylate (HBA), acetosyringone (AS), and violuric acid (VLA), which are oxidized by laccase and react with substrates through the hydrogen atom transfer route [57]. In the indirect catalytic process, chemoselective and mild oxidation of propargylic alcohols was performed by a combined reaction, which comprised the oxyradical TEMPO and laccase from *Trametes versicolor*. The same catalytic system was also used to oxidize 14 racemic alcohols [58]. Luo *et al*. reported that perfluorooctanoic acid was degraded due to electron loss, and that in the presence of HBT, Kolbe decarboxylation by laccase indirect catalysis produced a perfluoroheptyl radical [59].

Degradation regarding the indirect oxidation of laccase in lignin has attracted much attention. One reason for this is that the oxidation of lignin by laccase can induce the formation of H_2_O_2_, which is presumed to be a reaction between O_2_ and radicals from lignin [60]. Consequently, H_2_O_2_ can be used by lignin peroxidases (LiP) to degrade lignin. Another reason is that some intermediates, such as carboxyl radicals with strong oxidizing properties, can act as oxidants for LiP and manganese peroxidases (MnP) to initiate the enzymatic hydrolysis reaction after the direct oxidation of lignin [61].

### Applications of Laccase

Due to its high reduction potential, laccase can be used in various industrial fields, and many studies regarding laccase applications have been reported. The ability of laccase to depolymerize polymer materials makes it useful in biofuels, pulping, and papermaking [62, 63]. Regarding bioethanol production, because lignin binds the cellulose and hemicellulose and hampers the cellulose breakdown [64], its removal is a major part of that field. In the studies of biomass pre-treatment, many delignification methods have been used, and one of these achieved an 89% delignification yield by employing laccase [65]. In the pulp and paper industry, lignin is produced in the process of cooking and pulping as a co-product in pulp bleaching, or kraft pulping (approximately 130 million tons per year), in which lignin is combusted to produce electricity [66]. Bleaching of pulp often uses large amounts of various hazardous chemicals, such as chlorine and chlorine-based chemicals [67]. While several laccases from microorganisms have demonstrated the capacity to delignify, mechanical pulp or kraft pulp increases their brightness [68].

Laccase can directly oxidize substrates with the participation of oxygen molecules, and water is the only by-product, which makes them an ideal enzyme for the food industry. There are some phenolic compounds in fruit juice, beer, and red wine, and these affect the sensory characteristics and taste of the products [69]. Laccase can selectively oxidize phenolic substances to clarify products, maintain their flavor, and reduce the speed of discoloration and deterioration [70]. In addition, flour can be modified with laccase to enhance dough stability, reduce viscosity, and improve processability. For example, laccase makes baked goods like bread fluffy and even textured [71]. The protein in milk can be oxidatively cross-linked and gelatinized under the action of laccase, thereby changing the texture and flavor of the final product [72].

Laccase has been widely used in organic biosynthesis and biopolymer production due to its substrate polymerization ability [49, 73]. For instance, eight novel types of penicillin were obtained by the laccase-catalyzed reaction with four different ramifications of 2,5-dihydroxybenzoic acid and ampicillin/amoxicillin as the substrates [74]. Moreover, 16 novel cephalosporins were produced by the amination catalyzed by laccase [75]. Several small bio-molecules can also be formed by laccase-catalyzed reactions, including amino acids, antioxidants, and anti-inflammatory compounds [76-78]. In addition to the above, the application of laccase in polymer and organic chemistry continues to garner attention. Poly (2,6-dimethyl-1,4-oxyphenylene oxide), used as a high-performance engineering plastic, possesses remarkable chemical and physical performance, and was also produced by laccase from *P. coccineus* [79].

Laccase can be used in textiles, fibers, and bioremediation when its depolymerization and polymerization abilities are in synergy [80]. In these industrial processes, free radicals are produced by laccase via an oxidizing substrate and then polymerized to corresponding products. Moreover, the properties of protein-stabilized emulsions and gels are improved to maintain the desired texture [81], the color of the dyes is eliminated [82], and the toxicity of toxic substances is reduced or removed [83]. Bisphenol A is an endocrine disruptor that has been studied for many years with enzymatic biodegradation [84], and after being oxidized by laccase, some unknown high-molecular-weight compounds and other low-molecular-weight compounds, such as 4-isopropenylphenol, are formed [85].

Due to its electrochemical properties, laccase has also been used in biosensors. The coupling of laccases with electrodes and direct electron transfer is the key to realizing bioelectrocatalysis, which is one of the basic principles of biosensor devices [86]. A highly selective phenol biosensor based on laccase has been constructed [87]. The synergistic detection of required pollutants or products can be achieved by the tyrosinase-laccase system [88]. A new type of laccase biosensor was constructed by a Soft Plasma Polymerization technique, which has the advantages of high sensitivity and high regression coefficient for the determination of dihydroxybenzene isomers in water samples in a wide linear range [89].

## Strategies for Laccase Improvement

### Strain Screening and Laccase Characterization

Host strains are one of the decisive factors affecting laccase production, and many different methods have been developed to screen laccase-producing strains. The application of laccase indicators such as gallic acid, tannic acid, syringaldazine, guaiacol, 2,2-azinobis-(3-ethylbenz-thiazoline-6-sulfonate), Rimazol brilliant blue R (RBBR) and polymeric dye R-478 greatly facilitates the screening of laccase-producing strains, enabling them to be intuitively judged on solid medium [90]. Furthermore, with an increasing number of laccase genes being sequenced, metagenomics technology can be used to mine laccase genes in complex samples [91], while laccase genes can be conveniently located in the genome of laccase-producing strains [92]. A variety of strains with high laccase production ability were screened out, and the enzyme production conditions were optimized (Tables 2 and 3). Remarkably, although laccase uses oxygen as an electron acceptor, the enzyme has also been discovered in the anaerobic bacterium *Geobacter metallireducens* [7].

Apart from screening strains with high laccase production, it is also of great significance to screen laccases with different catalytic properties to cope with the challenges of complex catalytic environments in wide-ranging applications. For example, laccase as an electrode catalyst in a biofuel cell needs to withstand higher temperatures [108], and the application of low-temperature laccase can reduce the cost of heating the catalytic system. This type of laccase also needs to adapt to different catalytic pH values. In recent years, laccases with different optimal catalytic temperatures (ranging from 20°C-92°C) and pH values (from 2.0-10.0) have been isolated and characterized [109, 110]. Moreover, many laccases have been found capable of maintaining high enzymatic activity in catalytic systems that contain organic solvents, metal ions, and high salts concentrations, making them highly suitable in sewage treatment and soil remediation applications [111-113].

Since the discovery of laccase, many studies have been conducted on the selection of laccase-producing strains, the characterization of laccases, and the mining of new laccase genes. The screening of high laccase-producing strains has greatly improved the laccase production yield. The characterization and gene sequencing of laccases with different catalytic temperatures and pH values, salt tolerance, organic solvent resistance, etc., especially those with the above composite properties, have greatly expanded the number of laccase application scenarios. Although some laccases cannot be industrially produced due to low yield, high nutritional requirements, and strict production conditions, the characterization and structural identification of laccases with different characteristics provide a reference for the modification and design of laccases.

### Strain Mutagenesis for Increased Laccase Production

Mutagenesis is a powerful method to improve strain performance in the biotechnology industry. Many mutagenesis strategies such as UV, laser, and N^+^ ion implantation have been used in the physical and chemical methods to accelerate the evolution of laccase [114] (Table 4). To enhance laccase production, the mutant, high laccase-producing strain *P. ostreatus* UV-6 was obtained by UV radiation, and its activity was improved to 135 U/L, which was 77% higher than the wild-type strain. Mutant *Coriolopsis gallica* T906 was obtained by synergizing UV and chemical treatments; the gene transcription of the laccase significantly increased, and the laccase activity increased three-fold compared to the wild-type strain [115]. UV radiation and ethyl methane sulfonate were used to improve laccase production in *Fusarium incarnatum* LD-3, resulting in a five-fold increase in laccase activity when utilizing rice bran medium in tray fermentation [116]. To shorten the fermentation time, N^+^ ion implantation mutagenesis was used in *Ceriporiopsis subvermispora*, and the fermentation time of laccase by the mutant strain was 24 h shorter, and the laccase activity was 4.79 times higher than that of the original strain [117]. *Paecilomyces* sp. WSH-L07 was mutated by low-energy ion implantation and a genetically stable mutant S152 was obtained with laccase activity increased four-fold. Additionally, this mutant laccase was shown to be more active for a wider temperature range and pH than the native enzyme [118]. Although different mutagenesis methods can significantly improve the activity and characteristics of laccases, the regulatory mechanism of laccase production is still unclear, which greatly limits the mutagenic effect of strains.

## Cultivation Techniques for Laccase Production

### Submerged Fermentation

Submerged fermentation (SMF) is defined as fermentation in the presence of excess water under a rich-nutrition liquid medium. Industrial production of laccase is primarily accomplished by SMF, and has been achieved in 5-L, 500-L, and 5-ton reactors using *Pycnoporus* sp. SYBC-L3 [119].

Carbon and nitrogen sources are the main factors for the production of laccase by SMF. For laccase production, organic nitrogen sources are generally more effective than inorganic nitrogen sources [120]. The addition of organic nitrogen sources, from yeast extract, peptone, and malt extract to PDB medium, could significantly increase the laccase production of *P. ostreatus*, while the addition of inorganic nitrogen sources had no significant difference in enzyme production [108]. Simple carbon sources such as glucose, maltose, and cellobiose are commonly used in laccase production. The application of cellobiose instead of microcrystalline cellulose as a carbon source resulted in a 20-fold increase in the laccase activity of *Cerrena unicolor* [121], while another study also showed that *Trametes pubescens*, *C. unicolor*, and *T. versicolor* 775 can also efficiently utilize cellobiose as carbon source [122]. The optimal carbon source for laccase production is distinct in different microorganisms. The best carbon source of *Kluyveromyces* sp. Dw1 is glucose and the best carbon source of *Pichia* sp. Dw2 is maltose [123].

Using industrial and agricultural wastes as carbon and nitrogen sources in SMF not only effectively reduces production costs, it also improves the production of laccase. This is due to the lignin and other components in these raw materials, such as polyphenols in corn steep liquor and p-coumaric acid in wheat straw that can induce the expression of laccase [124, 125]. The laccase activity was increased to 42,000 ± 600 U/L from 12,000 U/L after adding food waste as carbon and nitrogen sources to the medium [126]. With diluted molasses distillery wastewater as the main nutrient, the laccase yield of *Coriolus hirsutus* was as high as 2,198.2 U/ml. Supplementation of 10 g/l of orange peel, tea, and bagasse in the basal medium of *Pleurotus ostreatus* increased laccase activity by 9-, 5-, and 2-fold, respectively, and the fermentation time was shortened [127]. Another study also showed that adding barley bran, grape straw, and grape seeds to the medium of *T. versicolor* increased the activity of laccase by 11, 6, and 4 times, respectively [128]. Using wheat bran and a small amount of glucose as carbon sources, the laccase activity of *C. unicolor* C-139 was as high as 416.4 U/ml at a 120-L scale. In a 10-L fermenter, the laccase of *Ganoderma lucidum* strain 447 achieved 188.6 U/ml using ethanol-production residue as the substrate [129].

Adding inducers to the culture medium is an important means to improve the production of laccase. Common inducers usually include metal ions, phenols, amines, and dyes. As a component of laccase, copper ions can improve laccase activity by regulating the transcription of the laccase gene in almost all fungi [130, 131]. The same strain can be induced by different inducers; for example, 16 kinds of aromatic phenols and 14 kinds of amine inducers, 4 kinds of recalcitrant dyes, and copper ion can significantly increase the laccase production of *Myrothecium verrucaria*. Meanwhile, the addition of most inducers delays the peak time of enzymatic activity, which may be caused by the toxic effect of inducers leading to a delay in the hyphal growth phase and the reduction of enzyme production during the early stage of fermentation. Almost all inducers have negative effects on the growth of microorganisms, and the self-protection response caused by this negative effect is also the basis of these inducers’ induction [124, 133]. Adding inducers at the later stage of fermentation is a coping strategy to reduce their negative impact on bacterial growth. For example, adding copper ions on the fourth day of fermentation of *Lentinus tigrinus* resulted in the highest laccase activity [134]. In addition, the synergistic effect of inducers can produce a stronger induction effect. When copper and 2,5-xylidine were added to the medium of *T. versicolor* at the same time, the laccase activity was 4.4 times and 8.2 times that of the control group, respectively [135]. In addition, the synergistic induction of copper and guaiacol or veratryl alcohol was also found in *Lentinus crinitus* [136]. Ethanol is also a commonly used inducer, and by adding ethanol to the medium of *P. cinnabarinus* ss3, the enzymatic activity of laccase was increased by an astonishing 155-fold [137].

Moreover, cultural conditions also have a significant effect on laccase activity by SMF. Temperature and pH have a significant effect on laccase production by fungi, with the suitable pH and temperature range being 4.5 to 6.0 and 25°C to 30°C, respectively [16]. Several statistical experimental designs, such as the Box-Benhken, Placket-Burman, Response Surface, Central Composite (CCD), and Hokes design, are widely used for the optimization of culture conditions to enhance laccase production. In general, low-speed stirring is more beneficial for laccase production, as excessive shear force may be detrimental to fungal hyphae growth and the stability of laccase. Moreover, Vaidya *et al*. show that inoculation in homogenized form in a stirred tank reactor can nearly double laccase production [138]. Certain physical factors may also affect or stimulate the production of laccase. Heat shock treatment increased the laccase activity of *T. versicolor* by more than 1.6-fold [139]. The laccase yield of *T. versicolor* mycelial culture treated by intermittent ultrasound was 1.8 times that of the control group [140]. In addition, Tween 80 is also reported to promote cell wall permeability to enhance extracellular laccase activity [141]. Operating in fed-batch culture improved the laccase production of *T. pubescens* by two-fold and achieved a laccase activity of 740 U/ml [122].

### Solid-State Fermentation

Solid-state fermentation (SSF) is a process in which microorganisms grow in an environment with very low or without free water. Usually, the growth rates of fungus in SMF are much faster than in SSF. Due to the excellent laccase-producing ability of fungi, especially white-rot fungi, production by fungal-based SSF has received increasing attention recently. SSF offers culture conditions that are similar to natural environments; it is advantageous for fungi growth and laccase production, and therefore simplifies downstream processing, such as enzyme separation and purification, which can save energy and reduce production costs [142].

Many kinds of agricultural wastes are rich in lignocellulose and lignin, which are excellent substrates for mycelium growth and laccase production in SSF [143]. Besides reducing substrate costs, there are some natural substances in certain lignocellulosic wastes that can act as inducers and enhance the laccase production in SSF [16]. Many natural lignocellulosic residues, such as rice straw, wheat bran, sunflower seed hulls, sawdust waste, tea residue, sugarcane bagasse, pistachio shell, *Borassus flabellifer* empty fruit bunch waste, and tomato waste have been successfully used in producing laccase by SSF [144-151]. Besides lignocellulosic wastes, some other agricultural by-products such as soybean cake are also used for laccase production [152]. Moreover, in some studies, the application of mixed substrates, such as wheat bran / corn straw, rice bran / rice husk, and banana peel / wheat bran in SSF has also achieved higher laccase yields [153-155]. *Trametes* sp. AH28-2 achieved the highest laccase production (2,100 U/g) with the medium containing 60% rape stem, 20% peanut shell, and 20% wheat bran [156].

Furthermore, besides the type of substrate, the particle size of the substrate is also a very important factor affecting laccase production. In addition to providing sufficient growth surface area, the appropriate substrate particle size should also meet the requirements of aeration and mass transfer during fermentation. In SSF by *T. versicolor*, the laccase activity of solid substrates with particle size > 500 μm was 1.4 times higher than that with particle size < 200 μm [157]. During SSF, the homogeneity of mycelium development, laccase distribution, and nutrition utilization were further aided by the ideal water retention value and increased enzymatic digestibility of the solid substrates that were produced by sodium hydroxide pretreatment. The greatest laccase production of 2,912.34 U/g was obtained from pretreated rice straw with a diameter less than 0.085 cm, which was 7.72 times higher than the control [158].

Supplementing of nutrients in SSF is also an effective way to increase laccase yield. Laccase production by fungi is usually affected by key nutritional factors such as carbon and nitrogen sources, inducers, and inorganic salts. When cultured in sugarcane bagasse supplemented with the mixed nitrogen source of 1 g/l peptone and urea, the activity of laccase in *L. crinitus* increased from 2,000 U/gds to 6,555 U/gds [103]. By adding 6 mg of CuSO_4_, 0.7 g of starch, and 0.16 g of yeast extract to 6 g of wheat bran, the laccase yield increased from 2,400 U/gds to 10,050 U/gds [101]. Supplemented nutrients can adjust the nutrient composition of the medium and be utilized more quickly, greatly improving the growth and enzyme production of microorganisms. The highest laccase activity of SSF reported so far was produced by *Pseudolagarobasidium acaciicola* AGST3, reaching 5.35×10^5^ U/g [106]. However, process control and scaling up are slow-paced in SSF, which causes a long fermentation time [159, 160].

### Co-Culture Systems

Numerous studies have shown that co-culture can significantly increase the enzymatic activity of laccase, and its mechanisms include nutrient competition, antagonistic action, self-protection, and induction of metabolites [161]. When *T. versicolor* was co-cultured with the non-laccase-producing strain *Candida* sp. HSD07A, glucose starvation caused by nutrient competition increased the laccase activity by 11.8 times [162]. Another study showed that in addition to glucose limitation, organic acids, ethanol, glycerol, and other metabolites in yeast can enhance the laccase production of *G. lucidum* [163]. Interestingly, yet another study showed that yeast metabolizes glucose to produce glycerol, and the relay of the second carbon source resulted in an increase in the secretion time of laccase, leading to a higher production of laccase [164]. Nutrient competition between *Sporidiobolus pararoseus* SSM-8 and *T. hirsuta* also resulted in about a 9.9-fold increase of laccase in *T. hirsuta* compared to monoculture [165]. In addition, *Gongronella* sp. W5 induces *Panus rudis* 25 times stronger than copper / O-toluidine inducer, and further studies show that the main induction effect is due to the pH-resistant metabolites produced by *Gongronella* sp. [166]. In SSF, the co-culture of *T. versicolor* and *A. niger* achieved a high laccase yield of 97,600 U/g [167]. The laccase activity of *C. unicolor* and *T. versicolor* co-cultured in a 7-L fermenter reached 476 U/ml [168].

### Genetic Engineering Strategies for Laccase Improvement

Industrial enzymes including laccase have normally been produced by recombinant organisms because native organisms are often inefficient for production, which is incompetent for large-scale application. The heterologous expression of laccases was achieved from various microorganisms in *E. coli*, such as *Streptomyces griseorubens*, *B. subtilis*, *B. clausii*, and *Cyathus bulleri* [169-172]. For instance, the laccase gene from *T. thermophilus* HB27 was expressed in *E. coli*. The optimal reaction temperature was 92°C, and the T_80_ was over 14 h [173]. However, the expression of laccase in *E. coli* often suffered from low levels because the recombinant laccase usually formed insoluble protein and lacked glycosylation [172, 174]. Therefore, a suitable host for laccase heterologous expression needs to be investigated [175]. Fungal laccase is a glycoprotein involved in various glycosylation processes, so yeast or fungi are suitable heterologous expression systems. However, the yield of heterologous expression of laccase in fungi and *S. cerevisiae* was not high [176]. The laccase from *T. versicolor* was expressed in *A. niger*, and enzyme activity of laccase with 2,700 U/L was achieved using minimal medium-containing sucrose and yeast extract [177]. In contrast, the laccase heterologous expression in *P. pastoris* was very successful [178]. Laccase from *P. ostreatus* was expressed in *P. pastoris* X33 and cultured in a 10-L bioreactor, which can produce laccase at 3,159.93 U/L in 8 days [179]. Promoters AOX1 and GAP are often used to express enzymes in *P. pastoris*, and higher laccase production was achieved using AOX1 promoter [180]. The high laccase production (140 U/ml) was achieved in a 2.5-L fermenter equipped with a methanol sensor system by *P. pastoris* SMD 1168 carrying the *lcc1* gene from *T. versicolor* [181]. A laccase encoded by the *lacD* gene from *T. versicolor* sp. 420 was expressed in the *P. pastoris* GS115, and obtained a higher yield of laccase at 83 U/ml [182]. Thereafter, the yield of laccase was further increased to 239 U/ml when heterologous expression in *P. pastoris* was conducted under high-density fermentation [183]. Usually, the homologous expression is slightly worse than the heterologous expression. The highest productivity of laccase by engineered *H. volcanii* US02 reached 170 U/ml with liquid medium [184]. The use of a dual promoter system (constitutive and inducible promoters) can improve the expression level of enzymes by enabling enzyme production throughout the entire growth period of the strain. Yadav *et al*. enhanced the expression of recombinant small laccase in *P. pastoris* by a double promoter system, and the maximal laccase activity of 120 U/ml was achieved after methanol induction [185]. However, the AOX1 promoter usually causes safety problems with methanol use and strict process control in large-scale fermentation. To avoid these problems, promoter replacement/modification, and metabolic modification have been implemented. The most successful method is that of Wang *et al*., who engineered a new methanol-free *P. pastoris* strain by altering AOX1 promoter transcription factors, which can be used for laccase production in the future [186]. In addition, the signal peptide has a significant impact on the secretory expression level of laccase [187]. Using a modified signal peptide can significantly enhance the secretion level of laccase in yeast and filamentous fungi [188, 189].

Moreover, excellent recombination techniques and well-established genetic manipulations, including directed evolution, site-directed mutagenesis, and DNA shuffling make yeast suitable for heterologous protein production [176]. The enzyme activity of laccase from *Basidio mycete* PM1 was enhanced 34,000-fold after eight rounds of evolution by mutagenic PCR and DNA shuffling while using *S. cerevisiae* as the host [190]. In addition to enzyme activity, other characteristics of laccase can also be improved, such as stability, alkali resistance, or substrate specificity. For industrial applications, alkaline-tolerant (pH >9.0) laccase is usually more preferred. Laccase from the *B. mycete* and *C. cinerea* Lcc9 which possesses optimum pH at alkaline conditions, was obtained after three rounds of evolution in *P. pastoris*. While the optimum pH of wild laccase was 6.5, a mutant named PIE5 showed an optimum pH of 8.5 and 8.0 against guaiacol and 2,6-DMP, respectively [191]. The variant MaL-M1 showed a 3-fold improvement with *K*_cat_ at pH 9.8 compared to wild-type laccase from *Melanocarpus albomyces* via sequence saturation mutagenesis [192]. The thermal stability of laccase from *Bacillus* sp. HR03 was increased by site-directed mutations, and the T_50_ of the variant was 3-fold higher than the native enzyme [193]. Bian *et al*. enhanced the ABTS oxidation activity by 104% and broadened the pH tolerance range (pH 3–9) of laccase mutant Y230R in *Streptomyces coelicolor*. Structural analysis revealed that this mutation reduced steric hindrance in the substrate-binding pocket and reorganized hydrogen bond networks. Additionally, improved hydrophobic packing in domain 2 contributed to increased thermostability [194]. Hao *et al*. used 6T1B from *Geobacillus stearothermophilus* as the template for homology modeling to construct single and double mutants of laccase-like (TrLac-like) enzyme. Among these, the A248D mutant exhibited an approximately 110-fold increase in catalytic efficiency compared to the wild type, while retaining thermostability [195]. Unlike those studies in which the enzyme property was improved in a single direction, Mateljak *et al*. improved the thermal and pH stability of laccase simultaneously. They screened the laccase mutant libraries *in vitro* using synthetic high-redox-potential mediators with different oxidation routes and chemical natures, and ran the computer-aided evolution experiments with guiding benchtop mutagenesis. The result showed that the T1-copper site of the evolved high-redox-potential laccase increased from 740 to 790 mV, with a concomitant improvement in thermal and acidic pH stability [196]. Some researchers also obtained laccases with enhanced antibiotic and industrial dye-degrading capabilities through a computer-aided screening strategy [197-199].

### Chemical Modification for Laccase Improvement

Immobilization is a mature method that promotes the reuse and separation of enzymes and maintains the most active conformation by "solidly" attaching the enzyme to a solid support [200]. Deska *et al*. classified five types of enzyme immobilization: covalent binding, adsorption, encapsulation, cross-linking, and entrapment [201]. Improving laccase by immobilization has been well studied. For instance, using bacterial cellulose as a carrier for laccase immobilization showed a high yield of laccase immobilization (>70%). The optimal pH and temperature of immobilized laccase were changed from 3 to 4 and 60°C to 70°C, respectively. This immobilized laccase remained at 65% of its original activity after 8 cycles [202]. The same carrier was used to immobilize laccase from *T. versicolor*, and the resulting product showed high catalytic activity and stability with a broader pH range compared to the free enzyme, and it retained 69% of the initial activity after 7 cycles [203]. Costa *et al*. used multi-walled carbon nanotubes (CNTs) as carriers to immobilize laccase, achieving 100% immobilization efficiency and 20.5% recovered activity in the CNTox-0.30-EN system, which kept its high performance over 5 cycles for oxidizing ABTS. The results showed that the system was successfully used for treating mixtures of four phenolic compounds, and the efficiency was similar to the free enzyme [204]. However, the laccase immobilizing system should be rigorously designed for use. For example, the decolorization efficiency of immobilized laccase to anthraquinone dye reactive blue 19 was decreased from 100% to 80% when the immobilized system was changed from epoxy-activated Sepabeads to magnetic carbon nanoparticles [205].

### Conclusions and Perspectives

Laccase is an excellent green enzyme that shows great potential in modern industry. This review summarizes the characteristics of laccase and the latest strategies for improving laccase production. Laccases from different sources display different functions, and should be properly screened before use. Effective classification of laccases and their mediators can facilitate application, while the continuous discovery of new production strains will further expand the laccase library. The remodeling method of laccase production has also become controllable and efficient. Protein engineering and immobilization have received increasing attention for improving the properties of laccases.

Although many promising results have been achieved concerning laccase production, further improvements are required to realize economical viability on an industrial scale. Bacterial/filamentous fungal heterologous expression hosts need to be further explored; safe production using methanol with *P. pastoris* is a challenge; model laccases have yet to be defined, and fewer laccases are compatible with cheap immobilized materials. Fortunately, the screening method for laccase-producing strains is mature. Mutagenesis of the production strains can appropriately reduce the fermentation time. The screening of safe promoters in *P. pastoris* has achieved good results. Several customized laccases with special functions such as thermostability have been reported and have good application prospects. Advancing the molecular engineering of laccase requires the development of a comprehensive structure–function database that integrates genetic sequences and biochemical characterization data of key functional domains. On this basis, AI-driven rational design models can be constructed. Such systems will guide an efficient sequential workflow, including source strain selection, identification of regulatory targets, and implementation of modification strategies, thereby accelerating the development of high-performance laccase variants. In the future, suitable bacterial/filamentous fungal heterologous expression hosts should be explored. The customization of model laccase needs to be further improved, and immobilized materials with high adaptability and low price are also very attractive for future development.

## Figures and Tables

**Table 1:** Overview of various species of laccases.

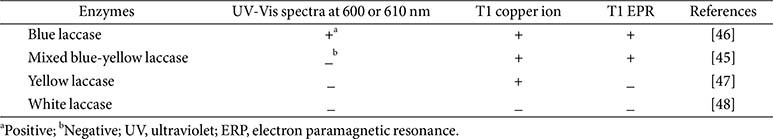

**Table 2:** Strains with high laccase yield in submerged fermentation.

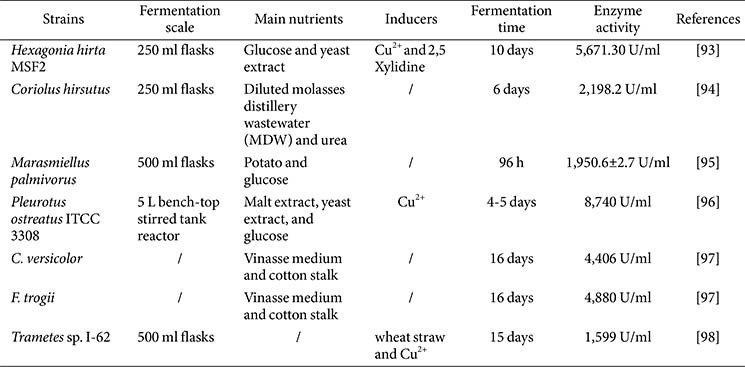

**Table 3:** Strains with high laccase yield in solid-state fermentation.

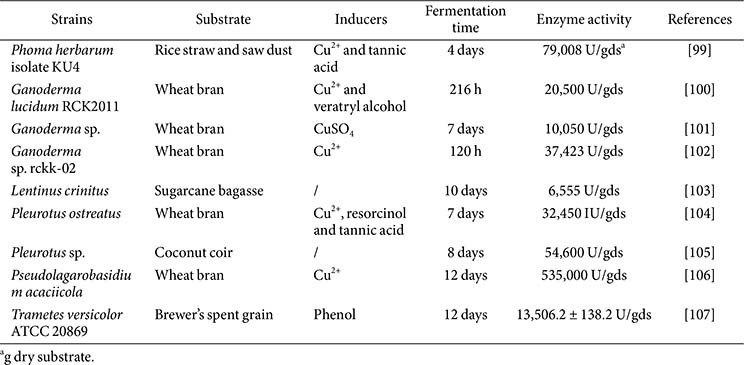

**Table 4:** Mutagenesis, heterologous expression, and directed evolution strategies to improve laccase production.